# *Sergentomyia* (*Neophlebotomus*) *chattiensis* n. sp.: morphological and molecular description of a new sand fly species from Himachal Pradesh, India

**DOI:** 10.3389/finsc.2026.1814368

**Published:** 2026-04-10

**Authors:** Harish Kumar Shah, P. A. Fathima, Manju Rahi, Prasanta Saini

**Affiliations:** ICMR- Vector Control Research Centre, Puducherry, India

**Keywords:** COI gene barcode, Himachal Pradesh, India, phlebotomine sand flies, *Sergentomyia (Neophlebotomus) chattiensis*

## Abstract

**Introduction:**

Himachal Pradesh, an ecologically diverse state in northern India, has recently emerged as a focus of atypical cutaneous leishmaniasis. As part of a molecular xenomonitoring, systematic entomological surveillance of sand flies resulted in the reporting of a novel species, *Sergentomyia (Neophlebotomus) chattiensis* n. sp. (Diptera: Psychodidae), from Chatti village in Kullu district, Himachal Pradesh, India.

**Methods:**

A systematic cross-sectional entomological survey was carried out in the districts of Kinnaur, Kullu, Shimla, and Mandi during August 2022, employing standard sand-fly collection techniques. Molecular characterization was performed using mitochondrial cytochrome c oxidase subunit I (COI) gene-based DNA barcoding, followed by phylogenetic analysis of the generated sequences.

**Results:**

The study reports *Sergentomyia (Neo.) chattiensis* as a newly recorded sand fly species and discusses its taxonomic association with other members of the subgenus *Neophlebotomus*. COI-based phylogenetic assessment confirmed that the collected specimens form a single taxonomic unit with negligible intraspecific genetic variation, while a genetic divergence of 12.3% from its closest congener supports its designation as a distinct species.

**Discussion:**

Despite its diverse physiography, rich biodiversity, and ecological suitability for sand fly breeding, Himachal Pradesh has lacked systematic entomological surveillance. The present study contributes to bridging this gap by expanding the existing knowledge of sand fly fauna in the state and providing comprehensive morphological and molecular characterization of this newly described species.

## 1 Introduction

Sand flies are small blood-feeding insects in the order Diptera and the family Psychodidae. They are known to spread several pathogens that significantly impact public health. They transmit *Leishmania* species, which cause different forms of leishmaniasis, *Bartonella bacilliformis*, the cause of Carrion’s disease, and Chandipura virus, which has been linked to outbreaks of Chandipura encephalitis (1). To date, about 1,060 valid sand fly species have been identified worldwide, and this number keeps growing as taxonomic studies and species discoveries continue (2). In India, researchers have steadily documented sand fly diversity. Currently, 72 species have been reported from various ecological and biogeographical regions of the country (3–6).

Himachal Pradesh also known as “Dev Bhumi” or the “Land of Gods” is located in the western Himalayan region of northern India, covers an area of approximately 55,673 km² and is characterized by diverse ecological and climatic conditions. The state has extensive forest cover, deep valleys, rugged mountain ranges, and varied agro-climatic zones, creating diverse ecological niches. Vegetation is dominated by pine and other forest species, and major river systems such as the Beas, Chenab, Yamuna, Satluj, and Ravi originate from this region, contributing to humid microhabitats (7).

The combination of these diverse geographical and climatic conditions, agricultural landscapes, and animal shelters associated with human dwellings in Himachal Pradesh creates favorable microhabitats that support the survival and proliferation of sand flies and other arthropod vectors, particularly in high-altitude environments. In recent years, the state has emerged as an important focus of leishmaniasis in India, with multiple indigenous cases of cutaneous leishmaniasis being reported (8–11). Additionally, molecular evidence confirming the presence of *Leishmania donovani* DNA has been reported from three sand fly species—*Phlebotomus longiductus*, *P. major*, and *P. bruneyi*—collected from the region (12). Despite these findings, sand fly diversity in Himachal Pradesh remains poorly explored, with only ten species classified under the genera *Grassomyia*, *Sergentomyia* and *Phlebotomus* documented so far (3, 12).

During a recent cross-sectional entomological survey conducted as part of molecular surveillance of leishmaniasis across four districts of Himachal Pradesh, Shah et al. (2024) (12) reported the discovery of a novel sand fly species, *Sergentomyia (Neophlebotomus) chattiensis* n. sp. The present communication provides a comprehensive morphological and molecular characterization of this newly described species, contributing to an in-depth understanding of sand fly diversity and vector ecology in the western Himalayan region.

## 2 Materials and methods

### 2.1 Study area

**Figure 1:**
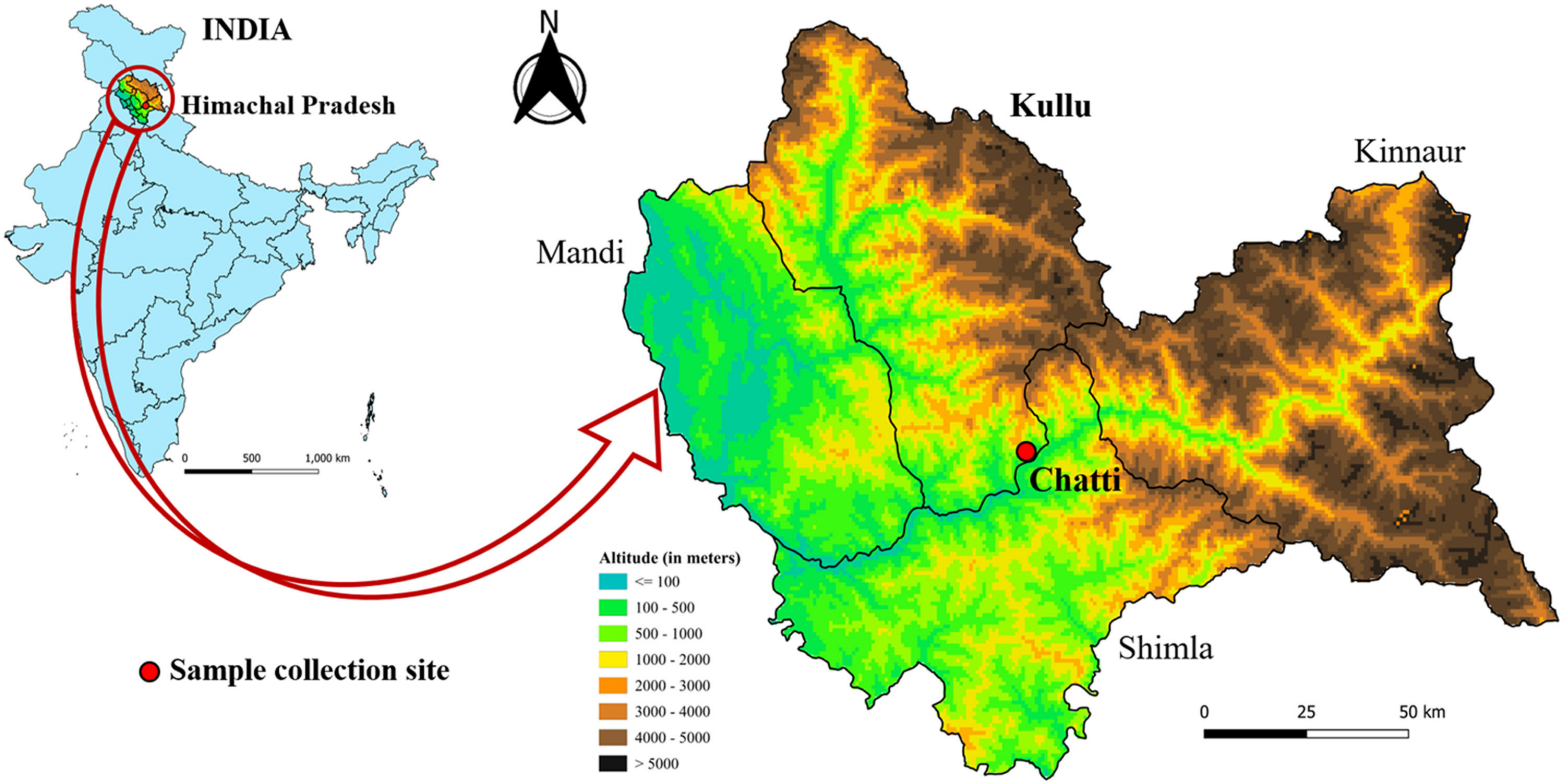
Sand fly specimen collection area in Chatti village of Kullu district in Himachal Pradesh, India.

A systematic cross-sectional entomological surveillance focusing on sand flies was carried out at seventeen sampling locations across four districts of Himachal Pradesh—Kinnaur, Kullu, Shimla, and Mandi—during August 2022 (**Figure 1**).

Kullu district, situated in the central Himalayan region, is widely recognized for its rich natural landscapes, cultural heritage, and traditional hospitality. Covering an area of approximately 547.71 km², the district supports a population of 437,903 people (13). The region’s cultural ethos, often referred to as Dev Sanskriti, represents a synthesis of mythology, spiritual traditions, and historical influences. Kullu is characterized by a pleasant climate and diverse terrain, encompassing the well-known towns of Kullu and Manali, the biodiversity-rich Great Himalayan National Park, the scenic Parbati Valley, distinctive hill architecture, and numerous trekking routes that attract visitors from across the world (13).

During August, Himachal Pradesh experiences the southwest monsoon, marked by episodic heavy rainfall, relative humidity levels exceeding 80%, and ambient temperatures ranging between 22 °C and 28 °C. Such climatic conditions are favorable for sand fly survival and breeding. Accordingly, sand flies were captured using standard entomological collection methods, including mechanical aspirators, CDC modified light traps, and sticky traps, following the protocol described by Alexander (2000) (14). Resting collections were conducted with aspirators between 0900 and 1200 h, while trap-based collections were operated from 1800 h to 0800 h the following day to cover peak crepuscular and nocturnal activity of sand flies.

### 2.2 Morphological identification of sand flies

A total of 443 sand fly specimens were collected from the region which comprises *Ph. argentipes* (2.48%), *Ph. longiductus* (41.99%), *Ph. burneyi* (17.16%), *Ph. major* (3.48%), *Ph. colabaensis* (0.45%), *Se. babu* (13.54%), *Se. kauli* (0.45%), *Sergentomyia* sp. (16.26%) and *Phlebotomus* sp. (3.84%). Sand fly specimens captured from different field locations were preserved in 70% ethanol and transported to the Indian Council of Medical Research–Vector Control Research Centre (ICMR-VCRC), Puducherry, for further processing. The specimens were dissected under a stereomicroscope (Stemi 305, Zeiss, Oberkochen, Germany) and mounted on glass slides using Hoyer’s medium. Morphological identification was carried out using a binocular microscope (Primostar 3, Carl Zeiss Suzhou Co., Ltd., China) following standard morphological identification taxonomic keys (15, 16).

A subset of both female and male specimens (14%; 62/443) exhibited distinctive morphological characters that did not correspond to any previously described sand fly species. These features clearly differentiated them from closely related members of the subgenus *Sergentomyia* (*Neophlebotomus*), particularly *Se. (Neo.) zeylanica* (Annandale, 1910) and *Se. (Neo.) arboris* (Sinton, 1931).

Detailed morphometric analyses (N = 10 of each female and male specimens) were conducted using a binocular microscope fitted with an ocular micrometer, and all measurements were logged in micrometers (µm). High-resolution digital images of key diagnostic characters were photographed using a microscope-mounted camera. Species description and morphological terminology followed the framework proposed by Galati et al. (2017) (17), and taxonomic nomenclature conformed to the International Code of Zoological Nomenclature (18). Morphometric data for a holotype female and allotype male along with 9 paratypes of *Se. (Neo.) chattiensis* n. sp. are provided in **Table 1**.

**Table 1:** Morphological parameters of female and male Sergentomyia (Neophlebotomus) chattiensis n. sp. (in µm).

Morphological parameters	Female (N = 10)				Male (N = 10)			
	Max	Min	Mean	SD	Max	Min	Mean	SD
Clypeus	209	176	190	14	165	154	158	6
Head length	550	400	480	35	490	400	438	32
Head width	450	400	430	17	480	400	439	27
Inter occular distance	200	160	177	14	180	150	164	12
Labrum	360	300	337	17	280	240	259	14
Teeth on hypopharynx	19	17	–	–	Rudimentary teeth			
No. of maxillary ventral teeth	26	23	–	–				
No. of maxillary lateral teeth	6	5	–	–				
Palpomere length P1	230	200	208	11	220	180	202	15
Palpomere length P2	220	180	202	12	210	160	196	16
Palpomere length P3	210	170	192	16	210	150	191	23
Palpomere length P4	150	120	142	10	240	100	185	37
Palpomere length P5	600	500	567	28	590	520	556	30
Newstead’s sensilla on P3								
Antennal flagellomere I (f1)	360	310	342	15	500	360	430	45
Antennal flagellomere II (f2)	143	121	129	6	180	161	173	8
Antennal flagellomere III (f3)	143	121	131	6	185	161	173	10
Ascoid on f2	55	51	53	2	48	40	45	2
Cibarial teeth	18	15	–	–	Rudimentary cibarial teeth			
Pharynx length	220	202	215	7	198	176	188	7
Pharynx Width	92	75	83	5	59	55	57	2
Pharyngeal armature	68	44	58	7	44	40	42	2
Wing length	2140	1926	2087	72	2140	1862	2080	105
Wing Width	749	642	683	35	663	556	616	35
Principal vein length								
Alpha	630	500	568	42	550	480	509	21
Beta	350	280	320	24	400	300	356	27
Gamma	430	330	373	28	450	340	387	34
Delta	440	380	403	21	400	330	358	23
R5	1669	1498	1591	49	1669	1434	1573	72
Fore leg								
Coxa	400	300	347	30	428	321	368	43
Trochanter	100	70	83	9	86	64	73	11
Femur	820	740	775	29	899	749	813	62
Tibia	1050	870	934	55	1177	963	1081	61
Tarsomeres								
T1	550	450	486	28	642	514	556	47
T2	270	230	253	14	278	214	253	20
T3	180	150	161	11	171	150	165	10
T4	150	130	135	7	150	128	139	11
T5	100	80	94	7	107	64	83	16
Length of spermatheca	77	70	74	3	–	–	–	–
Width of spermatheca	35	33	34	1	–	–	–	–
Length of common spermathecal duct	Common duct not visible				–	–	–	–
Length of spermathecal duct	Spermathecal duct not clear				–	–	–	–
Length of cerci	187	154	171	13	–	–	–	–
Genital furca	Not clearly visible				–	–	–	–
Sperm pump	–	–	–	–	140	120	128	8
Aedeagal duct	–	–	–	–	440	360	391	30
Sperm pump+Aedeagal duct	–	–	–	–	560	490	519	25
Ratio of Sperm pump/Aedeagal Duct	–	–	–	–	4	3	3	0
Paramere	–	–	–	–	210	160	180	20
Ejaculatory apodeme	–	–	–	–	110	100	105	5
Epandrial lobes	–	–	–	–	350	280	312	19
Gonocoxite	–	–	–	–	350	300	324	22
Gonostyle	–	–	–	–	220	200	209	10
Spine length	–	–	–	–	120	90	101	9
Length between terminal & terminal spines	–	–	–	–	130	100	117	11
Location of accessary spine from terminal spine	–	–	–	–	30	30	30	0
Tuft of hair	–	–	–	–	13	11	–	–

N, number of specimens considered for morphological analysis; Max, maximum; Min, minimum; SD, standard deviation; SC, sensilla chaetica; R, radius; T-tibia; ‘-’, not applicable.

### 2.3 Molecular identification

Whole genomic DNA (6 female and male specimens) was isolated from the preserved legs of individual sand fly specimens using the DNeasy Blood & Tissue Kit (Qiagen, Germany) in accordance with the manufacturer’s protocol, and the extracted DNA was finally eluted in nuclease-free water. For molecular identification and DNA barcoding, a ~720 bp region of the mitochondrial cytochrome c oxidase subunit I (COI) gene was amplified using PCR conditions and primers described by Kumar et al. (2012) (19). Bidirectional sequencing was outsourced and performed using the same primer sets, and the resulting nucleotide sequences were deposited in the National Center for Biotechnology Information (NCBI) GenBank database for public access and future reference.

### 2.4 Phylogenetic analyses

The obtained sequences were first compared with existing entries in the GenBank database using BLAST to identify their closest matches. Multiple sequence alignment was then carried out using MEGA version 11.0. Evolutionary relationships among the sequences were inferred by constructing a phylogenetic tree using the Neighbor-Joining (NJ) method based on the Kimura 2-parameter model, with branch support evaluated through 1,000 bootstrap replications. In addition, pairwise genetic distances and related statistical measures were calculated to assess levels of genetic divergence among the taxa.

## 3 Results

Family: Psychodidae Newman, 1834

Subfamily: Phlebotominae Rondani & Berté, in Rondani 1840

Genus: *Sergentomyia* França and Parrot, 1920

Subgenus: *Neophlebotomus* França and Parrot, 1920

**Figure 2:**
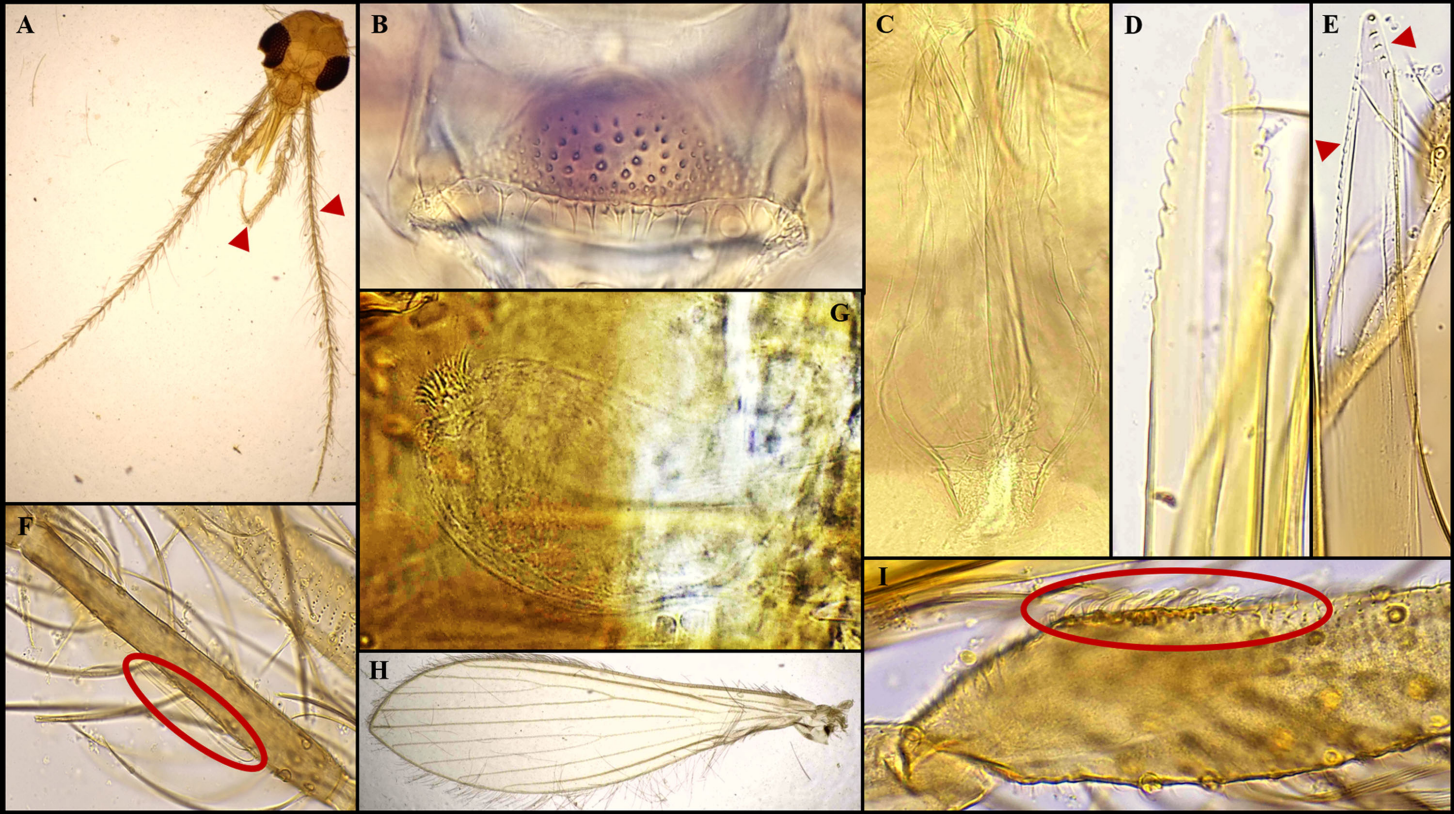
*Sergentomyia (Neophlebotomus) chattiensis* n. sp. (Female) **(A–I)**, **(A)** dissected head with 1- palps, 2- flagellomere; **(B)** cibarium with cibarial teeth and pigment patch; **(C)** pharynx; **(D)** hypopharynx; **(E)** 1- maxillary teeth (external and internal); **(F)** f2 with ascoid; **(G)** spermatheca; **(H)** Wing; **(I)** Newstead’s scales on p3.

**Figure 3:**
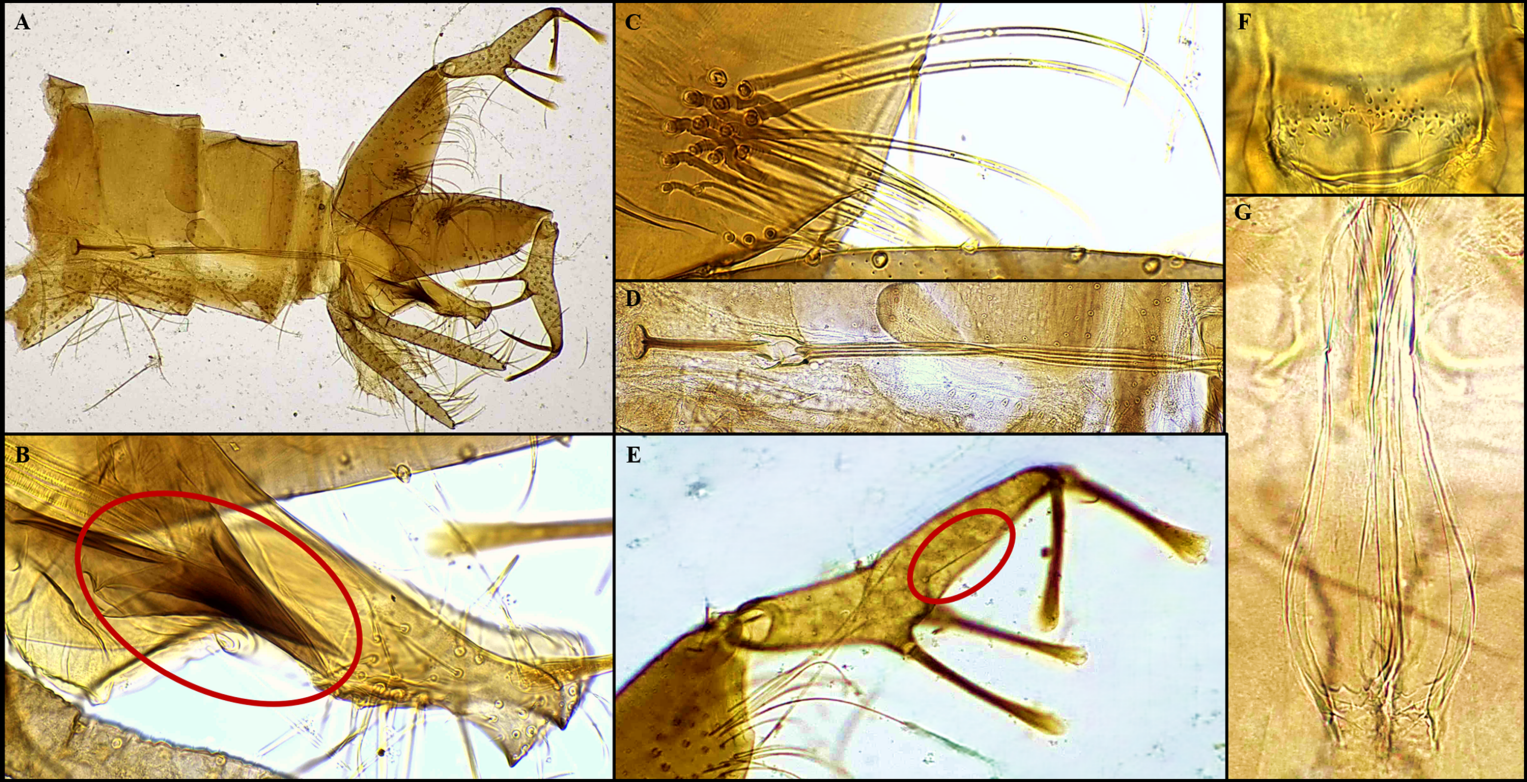
*Sergentomyia (Neophlebotomus) chattiensis* n. sp. (Male) **(A-G)**, **(A)** dissected terminalia; **(B)** aedeagus/parameral sheath and paramere with setae; **(C)** gonocoxite with setae on median tuft; **(D)** sperm/genital pump, filament/aedeagal duct; **(E)** gonostylus with spines along with accessory setae; **(F)** cibarium; **(G)** pharynx.

Species: *Sergentomyia (Neophlebotomus) chattiensis* n. sp. Shah et al., (**Figures 2**, **3**)

### 3.1 Female

#### 3.1.1 Holotype female

The specimen is light golden-brown in color. The head measures 430 µm in length and 480 µm in width, interocular distance is 177 µm. The labrum is 337 µm long. The hypopharynx bears approximately 17–19 teeth on each side. The maxilla is armed with 5–6 internal teeth and 23–26 external teeth. The palpal formula is 5,1,2,3,4 (p5 > p1 > p2 > p3 > p4), with palpomere lengths of p1 = 208 µm, p2 = 202 µm, p3 = 192 µm, p4 = 142 µm, and p5 = 567 µm. Approximately 25–30 club-shaped Newstead’s sensilla are present on the median region of palpomere 3; similar sensilla are absent on the remaining palpal segments. A single distal spiniform seta is observed on p3, three on p4, and eight on p5.

The antennal flagellomeres measure f1 = 342 µm, f2 = 129 µm, and f3 = 131 µm, with f1 longer than the combined length of f2 and f3. Each flagellomere from f1 to f13 bears a pair of ascoids that extend almost to the subsequent segment; the ascoid length on f2 is 53 µm. Simple setae are absent on flagellomeres f1–f13 but number 20–22 on f14. A single distal papilla is present on f1 and f2, absent from f3 to f9, followed by one papilla on f10 and f11, four on f12, five on f13, and eight on f14.

#### 3.1.2 Cibarium

The cibarium possesses a ventral plate with more than eight distinct rows of denticles (fore-teeth) and a dark, dome-shaped pigment patch on the dorsal plate, without a clearly defined anterior projection. A row of 15–18 well-developed, pointed cibarial teeth is present. The pharynx is pot-shaped, broader at the base, and lightly armed with minute spicules. It measures 215 µm in length and 83 µm in width, and pharyngeal armature depth is 58 µm, making it 2.6 times longer than wide.

#### 3.1.3 Wings

The wings are 2,087 µm long and 683 µm wide. The lengths of the principal vein sections are: alpha = 568 µm, beta = 320 µm, gamma = 373 µm, delta (R1 overlap) = 403 µm, and R5 = 1,591 µm. The wing index (alpha/beta) is 1.78.

#### 3.1.4 Fore leg

Measurements are as follows: coxa 347 µm, trochanter 83 µm, femur 775 µm, tibia 934 µm, and tarsomeres T1 = 486 µm, T2 = 253 µm, T3 = 161 µm, T4 = 135 µm, and T5 = 94 µm.

#### 3.1.5 Genitalia

The spermathecae are oblong, thin-walled, and terminate in a knob set within a shallow pit. Faint internal striations or wrinkles are visible throughout the spermathecal body, while the outer surface is smooth and lacks segmentation. Each spermatheca measures 74 µm in length and 34 µm in width and bears secretory cells at apical terminal end. Individual and common spermathecal ducts are often indistinct or not visible. The cerci are simple, measuring 171 µm in length. The genital furca is poorly developed and is indistinguishable (**Figure 2**).

### 3.2 Male

#### 3.2.1 Allotype male

The male specimen is light golden-brown in color, similar to the female. The head measures 438 µm in length and 439 µm in width; interocular distance is 164 µm; labrum length is 259 µm. Teeth on the maxilla and hypopharynx are poorly developed and not clearly distinguishable.

The palpal formula is alike to that of the female, 5,1,2,3,4 (p5 > p1 > p2 > p3 > p4). Palpomere lengths are p1 = 202 µm, p2 = 196 µm, p3 = 191 µm, p4 = 185 µm, and p5 = 556 µm. Approximately 15–20 club-shaped Newstead’s sensilla are present on the median region of palpomere 3 and are absent from the remaining palpal segments. A single distal spiniform seta is observed on p3, three on p4, and six on p5.

The antennal flagellomeres measure f1 = 430 µm, f2 = 173 µm, and f3 = 173 µm, with f1 longer than the combined length of f2 and f3. Each flagellomere from f1 to f13 bears a pair of ascoids extending nearly to the following segment; the ascoid length on f2 is 45 µm. Simple setae are absent on f1–f13 but number 18–20 on f14. One distal papilla is present on f1 and f2, absent from f3 to f9, followed by one papilla on f10 and f11, four on f12, five on f13, and eight on f14.

#### 3.2.2 Cibarium

The cibarium shows 14–17 faint, rudimentary cibarial teeth on the ventral plate and approximately eight rows of weakly developed denticles (fore-teeth). A pigment patch is present on the dorsal plate, though it is less pronounced compared to the female. The pharynx closely resembles that of the female, being pot-shaped with a slightly broader base and lightly armed with minute spicules. It measures 188 µm in length and 57 µm in width; pharyngeal armature depth is 41 µm, which is 3.3 times longer than wide.

#### 3.2.3 Wings

The wings measure 2,080 µm in length and 616 µm in width. The principal vein sections measure: alpha = 509 µm, beta = 356 µm, gamma = 387 µm, delta (R1 overlap) = 358 µm, and R5 = 1,573 µm. The wing index (alpha/beta) is 1.42.

#### 3.2.4 Fore leg

Measurements are as follows: coxa 368 µm, trochanter 73 µm, femur 813 µm, tibia 1,081 µm, and tarsomeres T1 = 556 µm, T2 = 253 µm, T3 = 165 µm, T4 = 139 µm, and T5 = 83 µm.

#### 3.2.5 Genitalia

The sperm pump measures 128 µm in length, while the aedeagal duct is 391 µm long, giving a combined length of 560 µm. The aedeagal duct is markedly longer than the sperm pump, with a sperm pump to aedeagal duct length ratio of approximately 1:3. The aedeagal filament is straight, slender, and distinctly striated throughout its length. The ejaculatory apodeme measures 105 µm. The gonocoxite is 324 µm long and bears a well-defined median tuft of 11–13 closely grouped internal setae. The gonostyle measures 209 µm in length and is armed with four robust spines—two apical and two terminal—with a characteristic rounded, spoon-like distal end measuring approximately 101 µm. The interspinal distance is 117 µm, and an accessory spine is located 30 µm proximal to the terminal spine. The paramere is beak-shaped, 180 µm long, and bears approximately 25–30 strong, upward-directed setae. The parameral sheath (aedeagus) tapers distally to a sharp, arrow-like point. The epandrial lobes measure 312 µm in length (**Figure 3**).

### 3.3 Diagnosis of *Sergentomyia (Neophlebotomus) chattiensis* sp. nov.

The presence of horizontal or recumbent/horizontal hairs on abdominal tergites II–VI in both sexes, together with the arrangement of cibarial teeth in a single row, the occasional presence of fore-teeth that are typically directed upwards, and a usually well-defined pigment patch, are diagnostic features of the genus *Sergentomyia*. Additional generic characters observed include a male style bearing four prominent spines along with an accessory seta. The cibarial teeth are generally parallel, often subequal in size, and not markedly narrow. The pharynx is slender and may be armed with teeth or scales, or occasionally nearly unarmed. Antennal flagellomere I is longer than the combined lengths of segments II and III. In females, the spermathecae are thin-walled and capsule-shaped, sometimes exhibiting faint internal striations, while in males the aedeagus is slender with a rounded/blunt tip and the paramere is distinctly hooked (**Figures 2**, **3**).

These shared and diagnostic morphological traits clearly support the placement of the present species within the subgenus *Neophlebotomus* of the genus *Sergentomyia*. In both sexes, the cibarium bears a row of 15–18 cibarial teeth, which are slightly less developed or more rudimentary in males. In females, the cibarial teeth are well defined and arranged in a row that tapers to a fine point, whereas in males the teeth are comparatively less distinct and poorly developed. In addition to the cibarial teeth, both sexes possess approximately eight rows of fore-teeth or small denticles, which are more clearly visible in females than in males. All these characters are consistently observed in the holotype, allotype, and paratype specimens of *Sergentomyia (Neophlebotomus) chattiensis* n. sp., confirming its taxonomic placement (**Figures 2**, **3**).

### 3.4 Morphological and molecular variability

**Figure 4:**
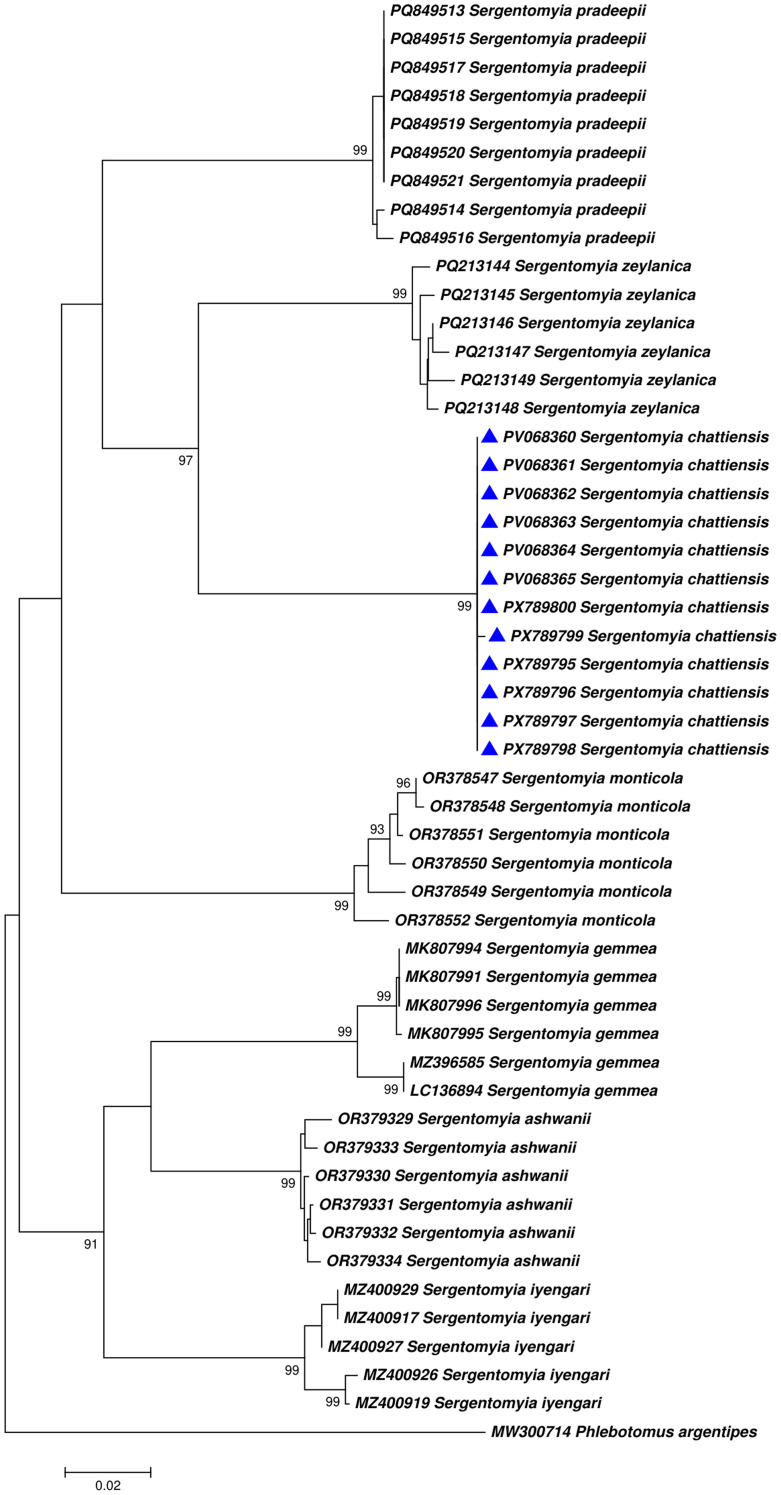
Phylogenetic analysis of mitochondrial Cytochrome *c* oxidase subunit I (COI) gene sequences for species of *Sergentomyia (Neophlebotomus) chattiensis* n. sp. along with *Se. (Neo.) pradeepii, zeylanica, ashwanii, gemmea, iyengari* and *monticola*; outgroup; *Ph. argentipes*.

The distinctive morphometric characters observed in the holotype, paratype female, allotype, and paratype male were consistent across all examined specimens (**Table 1**). All individuals of *Se. (Neo.) chattiensis* n. sp. were collected from the single habitat type within a single district and displayed uniform taxonomic features. DNA barcoding revealed identical COI sequences among all specimens, with no detectable nucleotide variation and a negligible Kimura two-parameter (K2P) genetic distance, confirming that they represent a single taxonomic unit. In contrast, the overall genetic divergence between *Se. (Neo.) chattiensis* n. sp. and its closest congener, *Se. zeylanica*, was 12.3% (**Figure 4**). Population genetic analysis performed using MEGA 7.0 indicated high genetic differentiation (*H_ST_* = 0.933) coupled with negligible gene flow (*N_m_* < 0.002). Together, these consistent morphological distinctions and molecular evidence clearly demonstrate that *Se. (Neo.) chattiensis* n. sp. represents a distinct and evolutionarily divergent species within the subgenus *Neophlebotomus*.

### 3.5 Type locality and materials

*Sergentomyia (Neophlebotomus) chattiensis* n. sp. accounted for approximately 14% of the total sand fly species composition recorded from Chatti village in Kullu district, Himachal Pradesh, India. The type locality for both the sexes paratype specimens comprises of cattle sheds and adjoining rooms in Chatti village, tehsil Nirmand, panchayat Tunan, Kullu district, Himachal Pradesh, India (31.4593° N, 77.6679° E; altitude 1,097 m above mean sea level).

Voucher specimens of the female holotype and male allotype were mounted on glass slides, assigned unique identification codes with complete collection data, and deposited in the museum of the Indian Council of Medical Research–Vector Control Research Centre (ICMR-VCRC), Puducherry-605006, India. Paratype specimens were deposited in the National Museum of the Zoological Survey of India (ZSI), Alipur, Kolkata, India. DNA sequences generated for molecular analysis were submitted to the National Center for Biotechnology Information (NCBI) GenBank under accession numbers PV068360–PV068365.

The type series comprises the female holotype (voucher GD-870 [VCRC]) and the male allotype (voucher GD-803 [VCRC]), both preserved at the ICMR-VCRC museum, Puducherry, India.

### 3.6 Etymology

The species *Sergentomyia (Neophlebotomus) chattiensis* n. sp. is named after Chatti village, the type locality from which the specimens were collected. Chatti is a small hamlet located in Nirmand Tehsil of Kullu District, Himachal Pradesh, India, and falls under the jurisdiction of Tunan Panchayat. The village lies approximately 85 km south of the district headquarters at Kullu, about 4 km from Nirmand, and nearly 69 km from the state capital, Shimla.

### 3.7 ZooBank registration

In accordance with Section 8.5 of the amended 2012 version of the ICZN (18), the details of the new species have been submitted to ZooBank. The associated Life Science Identifiers (LSIDs) are: urn:lsid:zoobank.org:pub:433C8BDA-7829-4097-8137-A009FE7CBCCE and urn:lsid:zoobank.org:act:C2F15E4C-E89A-4273-9BCF-53F0C5F969D1.

## 4 Discussion

A cumulative and updated checklist of sand flies from Himachal Pradesh, northern India, was previously compiled by Shah et al. (2023) (3). Building on this baseline, the current entomological surveillance, part of a nationwide molecular xenomonitoring program for leishmaniasis, focused on four districts in Himachal Pradesh: Kinnaur, Shimla, Kullu, and Mandi. Indigenous cases of leishmaniasis have been reported in these areas (9). These districts provide ideal conditions for sand fly breeding and survival during the pre-monsoon period, July to August. This time is marked by high humidity, intermittent rainfall, dense vegetation, plenty of vertebrate hosts for blood feeding, and varied physical features. Within this ecological setting, the present study documents the morphological and molecular characterization of the novel identified sand fly species, *Sergentomyia (Neophlebotomus) chattiensis* n. sp., from Chatti village in Kullu district.

The genus *Sergentomyia* França & Parrot comprises a large number of species grouped into several subgenera, among which *Neophlebotomus* is one of the most species-rich, with more than 21 species reported from the Oriental/Indomalayan region (2, 15, 16). To date, 20 species of *Sergentomyia* have been documented from India (3, 4, 6), including *Se. (Neo.) arboris*, *ashwanii*, *chakravarti*, *dhandai*, *gemmea*, *hodgsoni*, *hodgsoni hodgsoni*, *iyengari*, *kottamala*, *kurandamallai*, *linearis*, *malabarica*, *monticola*, *nilamburensis*, *perturbans*, *pradeepii*, *purii*, *quatei*, *verghesei*, and *zeylanica*. The present description of *Se. (Neo.) chattiensis* n. sp. adds to this growing list and further expands the known diversity of the subgenus in India.

Based on comparisons with standard taxonomic keys and original descriptions, specimens of *Se. (Neo.) chattiensis* n. sp. show affinities with closely related congeners such as *Se. (Neo.) zeylanica* and *Se. (Neo.) arboris* (15, 16, 20). However, several consistent morphological differences clearly distinguish the new species. Females of *Se. zeylanica* typically possess 14–15 cibarial teeth, three rows of fore-teeth, a dome-shaped pigment patch, and thin-walled, striated spermathecae; the labrum measures approximately 310 µm, and the maxilla bears three lateral and about thirty ventral teeth. In contrast, females of *Se. arboris* are characterized by around eight rows of cibarial fore-teeth, 17–20 cibarial teeth, and the absence of a clearly defined anterior process on the pigment patch; the labrum measures about 320 µm, and the maxilla bears eight lateral and nearly 58 ventral teeth. Females of *Se. (Neo.) chattiensis* n. sp., however, are distinguished by the presence of 15–18 well-defined, tapering cibarial teeth arranged in a row, approximately eight rows of fore-teeth, and a dome-shaped pigment patch lacking a distinct forward process. The spermathecae are oblong, thin-walled, and exhibit faint wrinkles or striations, terminating in a short knob set within a pit—features that clearly separate this species from its closest congeners.

Male specimens also show clear diagnostic differences. Males of *Se. zeylanica* possess an irregular and poorly defined arrangement of 8–10 cibarial teeth and a faintly visible pigment patch, along with a relatively thick aedeagus and a gonocoxite bearing clusters of outer hairs. In *Se. arboris*, the cibarium bears about ten prominent fore-teeth and roughly twenty small rounded teeth, with the pigment patch absent or poorly developed; the aedeagus is triangular with a blunt tip. In contrast, males of *Se. (Neo.) chattiensis* n. sp. exhibit 14–17 rudimentary but discernible cibarial teeth, approximately eight rows of weakly developed fore-teeth or denticles, and a less prominent pigment patch on the dorsal plate. Although the general arrangement of spines on the gonostyle is similar across congeners, *Se. chattiensis* males are uniquely characterized by a well-defined median tuft of 11–13 internal setae on the gonocoxite, a feature that reliably differentiates them from related species.

Molecular characterization using COI DNA barcoding further corroborates the taxonomic distinctness of *Se. (Neo.) chattiensis* n. sp. All the analyzed specimens showed identical sequences with no detectable nucleotide variation. This resulted in negligible genetic distance within the species. In contrast, the overall genetic difference between *Se. chattiensis* and its closest related species, *Se. zeylanica*, was 12.3%. A population genetic study using MEGA 7.0 showed high genetic differences (*H_ST_* = 0.933) and very low gene flow (*N_m_* < 0.002). These consistent physical differences and strong molecular evidence confirm that *Se.* (*Neo.*) *chattiensis* n. sp. is a distinct and evolutionarily separate species within the subgenus *Neophlebotomus*.

## 5 Conclusions

The present study provides a comprehensive morphological and molecular characterization of *Sergentomyia (Neophlebotomus) chattiensis* n. sp., a novel sand fly species from Himachal Pradesh, India. This record raises the known sand fly fauna of the state from 10 to 11 species. The findings highlight the importance of sustained and systematic entomological surveillance to improve understanding of sand fly diversity in the region and to assess their possible role in disease transmission.

## 6 Limitation

The molecular analysis in this study was performed using a single mitochondrial COI marker, which is widely used for species identification and molecular characterization. Future studies incorporating multiple genetic markers may further enhance the understanding of evolutionary relationships among sand fly species.

## Data Availability

The datasets presented in this study can be found in online repositories. The names of the repository/repositories and accession number(s) can be found in the article/supplementary material.
